# Actions of L-thyroxine (T4) and Tetraiodothyroacetic Acid (Tetrac) on Gene Expression in Thyroid Cancer Cells

**DOI:** 10.3390/genes11070755

**Published:** 2020-07-07

**Authors:** Paul J. Davis, Hung-Yun Lin, Aleck Hercbergs, Shaker A. Mousa

**Affiliations:** 1 Department of Medicine, Albany Medical College, Albany, NY 12208, USA; 2 Pharmaceutical Research Institute, Albany College of Pharmacy and Health Sciences, Rensselaer, NY 12144, USA; shaker.mousa@acphs.edu; 3 Ph.D. Program for Cancer Molecular Biology and Drug Discovery, College of Medical Science and Technology, Taipei Medical University, Taipei 11031, Taiwan; linhy@tmu.edu.tw; 4 Cancer Center, Wan Fang Hospital, Taipei Medical University, Taipei 11031, Taiwan; 5 Traditional Herbal Medicine Research Center of Taipei Medical University Hospital, Taipei Medical University, Taipei 11031, Taiwan; 6 Department of Radiation Oncology, The Cleveland Clinic, Cleveland, OH 44195, USA; hercbergs@gmail.com

**Keywords:** apoptosis, angiogenesis, integrin αvβ3, L-thyroxine (T4), thyroid cancer, tetraiodothyroacetic acid (tetrac)

## Abstract

The clinical behavior of thyroid cancers is seen to reflect inherent transcriptional activities of mutated genes and trophic effects on tumors of circulating pituitary thyrotropin (TSH). The thyroid hormone, L-thyroxine (T4), has been shown to stimulate proliferation of a large number of different forms of cancer. This activity of T4 is mediated by a cell surface receptor on the extracellular domain of integrin αvβ3. In this brief review, we describe what is known about T4 as a circulating trophic factor for differentiated (papillary and follicular) thyroid cancers. Given T4′s cancer-stimulating activity in differentiated thyroid cancers, it was not surprising to find that genomic actions of T4 were anti-apoptotic. Transduction of the T4-generated signal at the integrin primarily involved mitogen-activated protein kinase (MAPK). In thyroid C cell-origin medullary carcinoma of the thyroid (MTC), effects of thyroid hormone analogues, such as tetraiodothyroacetic acid (tetrac), include pro-angiogenic and apoptosis-linked genes. Tetrac is an inhibitor of the actions of T4 at αvβ3, and it is assumed, but not yet proved, that the anti-angiogenic and pro-apoptotic actions of tetrac in MTC cells are matched by T4 effects that are pro-angiogenic and anti-apoptotic. We also note that papillary thyroid carcinoma cells may express the leptin receptor, and circulating leptin from adipocytes may stimulate tumor cell proliferation. Transcription was stimulated by leptin in anaplastic, papillary, and follicular carcinomas of genes involved in invasion, such as matrix metalloproteinases (MMPs). In summary, thyroid hormone analogues may act at their receptor on integrin αvβ3 in a variety of types of thyroid cancer to modulate transcription of genes relevant to tumor invasiveness, apoptosis, and angiogenesis. These effects are independent of TSH.

## 1. Introduction

The clinical behavior of thyroid gland cancers is seen to reflect gene mutation and/or epigenetic changes [1,2], and effects of circulating or local trophic factors [3,4]. Circulating trophic factors include target tissue-specific thyrotropin (TSH) secreted by the pituitary gland and adipose tissue-source leptin, which enhances growth of a variety of tumors, including those of the thyroid gland [5]. In the clinical management of differentiated thyroid cancers, host pituitary TSH secretion is suppressed with exogenous L-thyroxine (T4) in conjunction with tumor surgery and radioablation of the cancers. 

Discovery of a cell surface thyroid hormone analogue receptor on plasma membrane integrin αvβ3 has provided additional information about the biological activity of T4. T4 has been viewed primarily as a source of 3,3′,5-triiodo-L-thyronine (T3), the principally active form of the hormone at nuclear thyroid hormone receptors (TRs) [6,7]. At physiological concentrations, however, T4 is the primary ligand of the cell surface iodothyronine receptor on the plasma membrane [8]. The integrin is generously expressed by cancer cells and rapidly dividing endothelial cells [6] and at this site, T4 promotes proliferation of tumor cells and supports angiogenesis [8]. T4 may also be a factor that contributes to metastasis of cancer cells [9]. 

Reverse T3 (3,3′,5′-triiodo-L-thyronine, rT3) has also been thought to have little bioactivity but is known to affect the state of actin in cells [10] and, recently, to stimulate proliferation of cancer cells [11]. In contrast to T4 and to rT3, tetraiodothyroacetic acid (tetrac), a derivative of T4, has anti-proliferative and anti-angiogenic properties at αvβ3 [6,8,12]. Prior to recognition of anti-tumor activity of tetrac at the cell surface integrin, tetrac was seen to have low-grade T3-like activity at the nuclear receptors [6]. 

**Figure 1:**
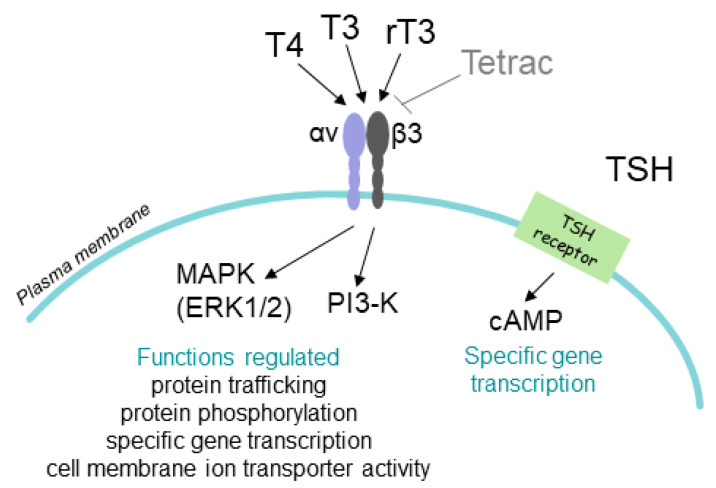
Cancer-relevant actions of thyroid hormones and pituitary thyrotropin (TSH) at thyroid carcinoma cells. Plasma membrane integrin αvβ3 contains a cell surface receptor for thyroid hormones and is overexpressed by cancer cells. T4 is the principal ligand for this receptor and the T4 signal is transduced by mitogen-activated protein kinase (MAPK/ERK1/2) or phosphatidylinositol 3-kinase (PI3-K) into cancer-linked gene transcription. A deaminated analogue of T4, tetrac, blocks actions of T4 at the integrin and is under development as an anticancer agent. At physiological concentrations, T3 is not active at the integrin, but reverse T3 (rT3) has cancer cell-stimulating activity at this plasma membrane receptor. T3 is the principal ligand of nuclear thyroid hormone receptors (TRs) in normal cells; TRs are not shown in the figure. Pituitary TSH acts at a specific cAMP-generating receptor on the plasma membrane of thyroid cancer cells. TRs and the TSH receptor are not structurally related to the T4 binding site on αvβ3. 2. T4 Actions at the Integrin αvβ3 in Papillary and Follicular Thyroid Carcinoma Cells.

Against this background, we examine in the present review the actions of T4 and tetrac, and chemical derivatives of tetrac on the biology of human thyroid cancer cells, including expression of a number of genes relevant to proliferation, apoptosis, and angiogenesis. These actions of T4 and tetrac are initiated at the hormone receptor on integrin αvβ3 (Figure 1). The signals generated at the integrin by thyroid hormone analogues involve early transduction within the cell primarily by mitogen-activated protein kinase (MAPK) [6,8].

## 2. T4 Actions at the Integrin αvβ3 in Papillary and Follicular Thyroid Carcinoma Cells

The physiological loops that connect the components of the normal hypothalamic-pituitary-thyroid gland axis have been assumed to be intact when the axis is disrupted by thyroid cancer [3,6,13,14,15]. In differentiated thyroid cancers, the tumor cells are usually TSH-responsive in terms of proliferation and pharmacologic administration of thyroid hormone—as T4, serving as a source of T3 at the level of nuclear TRs in the pituitary—and can take advantage of the thyro-pituitary feedback loop and suppress endogenous thyrotropin. The reduction of circulating TSH frequently contributes to arrest of the thyroid cancer. The amounts of exogenous T4 that are required to fully suppress host TSH via generated T3 are, by the nature of the definition of the feedback loop, supraphysiologic. The thyroid hormone receptors involved are exclusively nuclear TRs.

**Figure 2:**
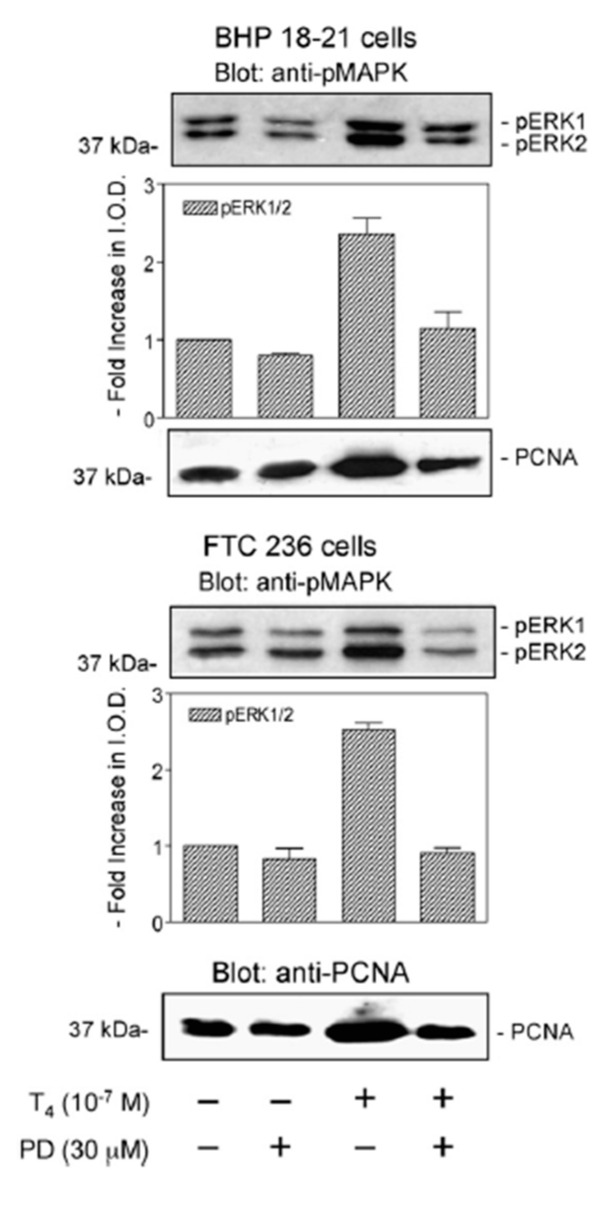
Proliferative activity of T4 in vitro on differentiated human papillary (BHP 18-21; upper panel) and follicular (FTC 236: lower panel) thyroid carcinoma cells. Proliferation was measured by immunoblotting of proliferative cell nuclear antigen (PCNA) from cultured cells. The 2.5-fold increase in PCNA in both cell lines was achieved with 10^−7^ M total T4 concentration (10^−10^ M free T4 in medium) [17]. The proliferative effect of the hormone required activation of ERK1/2 (MAPK). Inhibition of activation of ERK1/2 with PD98059 (PD) prevented enhancement of proliferation in both cell lines. T4 activates MAPK via the thyroid hormone analogue receptor on the extracellular domain of plasma membrane integrin αvβ3 [8,17]. Reprinted with permission from Elsevier from Lin et al. [16]. ERK1/2, extracellular signal-regulated kinases 1 and 2; MAPK, mitogen-activated protein kinase.

Lin et al. [16] showed in 2007 that differentiated papillary and follicular human thyroid carcinoma cells in vitro proliferated in response to physiological levels of T4. The index of cell division was expression of proliferating cell nuclear antigen (PCNA) (see Figure 2). The proliferative effect of T4 was inhibited by tetrac that, as noted above, blocks actions of T4 that are initiated at integrin αvβ3. An Arg-Gly-Asp (RGD) peptide that acts on a number of integrins and has a receptor close to the T4 receptor on αvβ3, also blocked the action of T4 on thyroid cancer cells, but a control Arg-Gly-Glu (RGE) peptide did not affect the action of T4. This report, thus, documented a local and primary effect of the principal hormonal product of the thyroid gland on thyroid gland cancer cells. TSH was not involved. The cell lines in this work had been well-studied by other endocrine laboratories and were the subjects of more than 20 publications. 

Another feature of the Lin et al. study was to define molecular components of the mechanism, downstream of its receptor on the integrin [8,16,17] by which T4 stimulated cell proliferation. That activation of MAPK was essential to the induction of proliferation was shown with the use of pharmacologic inhibitor of MAPK [18] (Figure 2), which eliminated the stimulatory T4 action on PCNA. The requirement for MAPK participation in T4′s action on proliferation has been shown in a number of cancer cells [19,20,21,22,23,24,25]. Only at supraphysiologic concentrations was T3 effective as a proliferative factor [16] and this is a feature of cancer cell responses that are mediated by the receptor for thyroid hormone on integrin αvβ3 [17]. 

**Figure 3:**
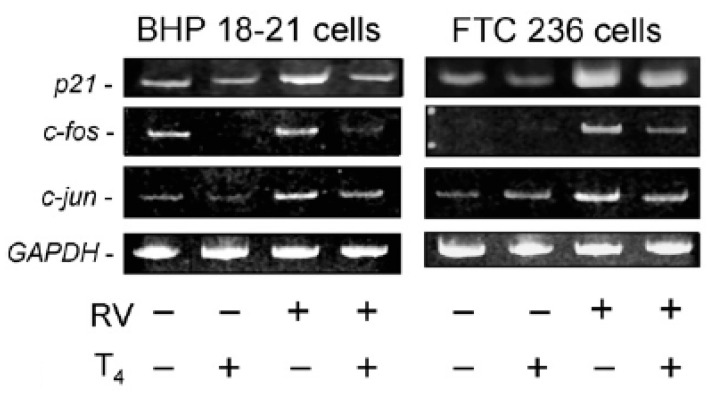
Effects of T4 on mRNA abundance (RT-PCR) of pro-apoptotic *p21*, *c-fos*, and *c-jun* in human differentiated papillary (BHP 18-2) and follicular (FTC 236) thyroid carcinoma cells. Cells were treated in vitro for 24 h with resveratrol (RV) (10 µM) or T4 (10^−7^ M total hormone concentration, 10^−10^ M free hormone) or with both agents. T4 inhibited the expression of RV-induced pro-apoptotic genes and did not affect control *GAPDH* gene. *GAPDH*, glyceraldehyde 3-phosphate dehydrogenase. Reprinted with permission from Elsevier from Lin et al. [16].

To define the specific genes whose transcription is affected by T4, Lin and co-workers [16] studied the effects of T4 on pharmacologically induced apoptosis in papillary and follicular thyroid cancer cells. The stilbene, resveratrol, induces apoptosis in differentiated thyroid cancer cells by a complex molecular mechanism that involves *p21*, *c-Jun*, and *c-Fos* expression [26]. As shown in Figure 3, resveratrol activated pro-apoptotic p53 and increased cancer cell expression of *p21*, *c-Fos*, and *c-Jun* genes. Addition of T4 to the cells cultured with resveratrol prevented p53 activation and apoptosis and also blocked induction of expression of *p21*, *c-Jun*, and *c-Fos*. Thus, T4 has anti-apoptotic activity in thyroid cancer cells by multiple mechanisms, including expression of multiple genes and reversing activation of p53. It is also important to note that tetrac inhibited resveratrol-induced apoptosis, as shown in nucleosome ELISA studies [16]. The anti-apoptosis effects of T4 in these studies, like those on cell proliferation, are initiated at the cell surface thyroid hormone receptor on integrin αvβ3.

Poorly differentiated or anaplastic thyroid carcinoma cells were not studied by Lin et al. in terms of possible responsiveness to T4.

## 3. Gene Expression in Thyroid Cancer Cells Exposed to Leptin

In the clinical setting of overweight, adipose tissue is expected to secrete leptin protein [27,28]. An endogenous anti-appetite factor, leptin has also been shown to support the growth of certain tumors, including papillary thyroid carcinoma, that express the leptin receptor [4]. There are a variety of other observations that link leptin and thyroid hormone together at cancer cells. Thyroid hormone may increase adipocyte secretion of leptin [29], and circulating leptin levels may be increased in papillary thyroid cancer patients [30]. Leptin variably modulates iodide uptake by thyroid cells [31]. Migration in vitro of papillary thyroid carcinoma cells is increased by leptin [32]. Finally, stimulation of epithelial-to-mesenchymal transition (EMT) is obtained with leptin [33] and with thyroid hormone [34], suggesting that both factors may support cancer metastasis.

**Figure 4:**
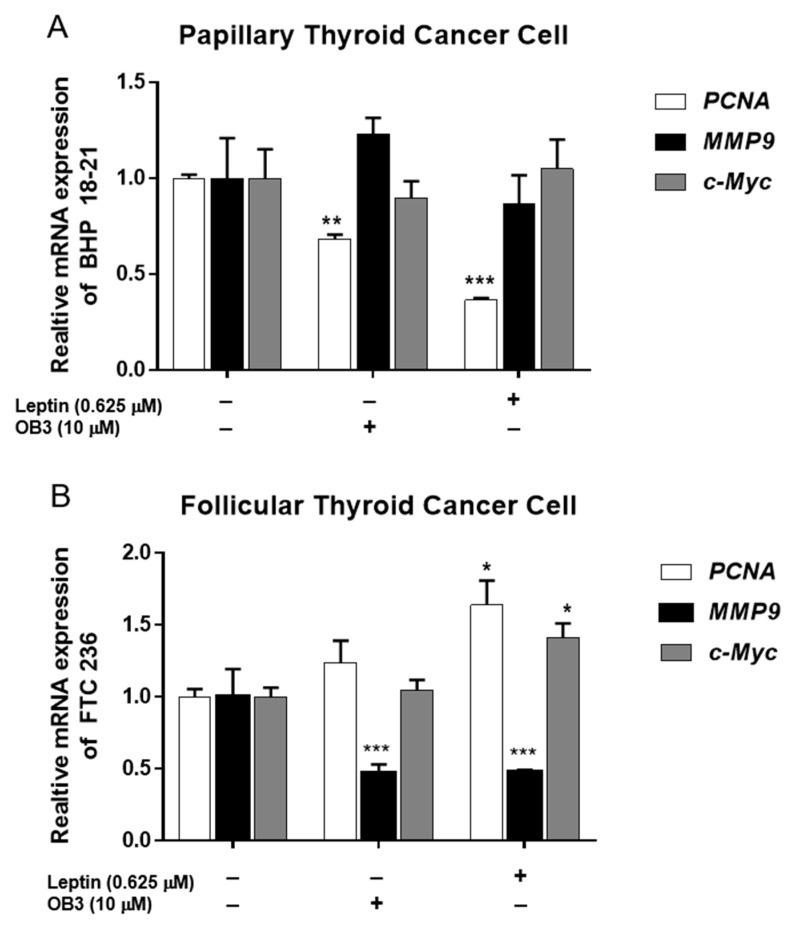
Effect of OB3 and leptin peptides on expression of genes relevant to invasion and cell proliferation in thyroid cancer cell lines. (**A**) Papillary and (**B**) follicular thyroid cancer cells were treated with either 0.625 µM leptin or 10 µM OB3 for 24 h. Cells were harvested, and total RNA was extracted. qPCR for *PCNA*, *MMP9* and *c-Myc* was conducted as described in Yang et al. [5]. Data were expressed as mean ± S.D. in triplicate. * *p* < 0.05, ** *p* < 0.01, *** *p* < 0.001, were compared with control.

Lin and co-workers have identified certain genes whose transcription is modulated in papillary thyroid carcinoma cells by leptin (Figure 4) [5]. Leptin and its derivative, OB3, both significantly reduce abundance of MMP9 mRNA in follicular thyroid cancer cells (Figure 4B), but do not affect expression of *MMP9* in papillary thyroid carcinoma cells (Figure 4A). In the latter cells, however, OB3 and leptin both reduce cell proliferation. Thus, leptin has thyroid cancer-type-specific actions on gene transcription. Such observations raise the possibility that body mass index with changes in endogenous leptin production may be associated with different clinical behaviors of papillary vs. follicular thyroid carcinomas.

## 4. Actions of Thyroid Hormone Analogues on Cells of Medullary Thyroid Carcinoma (MTC)

Medullary carcinoma originates in thyroid gland C cells, is thus distinct from papillary and follicular thyroid cancers and is an aggressive, non-TSH-dependent form of thyroid cancer. MTC may occur sporadically or conjunctively as a component of genetic multiple endocrine adenomatosis type 2 [35]. 

Gene expression that is subject to regulation in MTC cells by certain thyroid hormone analogues has been studied by Yalcin and co-workers [36]. In these studies, tetrac was the thyroid hormone analogue used to probe for thyroid hormone sensitivity of gene expression. Because tetrac, in various non-thyroidal cancer cells studied to-date, is anti-proliferative, pro-apoptotic, and anti-angiogenic [8,37]—the antithesis of T4 [19,21,22,23,24,38]—several implications of tetrac studies are clear. First, when it may be studied in the same cells, T4 is likely to have effects that are the direct opposite of tetrac and chemically modified tetrac. Tetrac is an antagonist of T4 at the integrin. Second, the panel of tetrac effects conveys the prospect of effectiveness as an anticancer agent. Third, the effects downstream on a) signal transduction and b) consequent gene expression of the αvβ3 thyroid hormone receptor are extensive.

Acting on MCT cells grafted into the chick chorioallantoic membrane (CAM) model system, tetrac or tetrac analogues reduced tumor weight and tumor hemoglobin content—an index of angiogenesis—by more than 60% at 3 weeks [36]. This anti-angiogenic effect was then studied with q-PCR and RNA microarray in cultured MTC cells. Here, tetrac or a formulation of tetrac downregulated expression of vascular endothelial growth factor A (VEGFA) and upregulated anti-angiogenic thrombospondin 1 (*THSB1*) genes. The tumor shrinkage in the CAM studies reflected anti-angiogenesis, but in addition pro-apoptosis gene expression was observed. For example, significant increases in transcription of *DFFA*, *FAF1* and *CASP2* were induced by tetrac molecules. Thus, thyroid hormone analogues have access to control mechanisms via αvβ3 for MCT angiogenesis- and apoptosis-relevant gene expression. Because tetrac is a specific inhibitor of the actions of T4 at integrin αvβ3, we propose that T4 has actions in MCT cells that are anti-apoptotic, as has been shown to be the case in papillary and follicular thyroid cancer cells.

## 5. Discussion

Clinical activity of the various forms of thyroid carcinoma is largely a function of specific gene mutations and epigenetic changes that are the subjects of other papers in this issue of the journal. Endogenous TSH may be a trophic factor, particularly in differentiated thyroid cancers and, as pointed out above and elsewhere in this symposium, pharmacologic administration of T4 to suppress host pituitary TSH production may be therapeutically helpful. When differentiated thyroid carcinomas recur in the context of T4-conditioned suppression of TSH, we have suggested that the tumors are no longer TSH-dependent and, in fact, may be T4-dependent [3]. This possibility has not been systematically examined. 

T4 is a growth factor for human carcinomas, including thyroid cancers, and a number of studies have concluded that a receptor for thyroid hormone analogues is involved on the extracellular domain of cancer cell integrin αvβ3 [8,37,39,40]. Transduction of the T4 or other thyroid hormone analogue signal at the integrin results in the downstream modulation of transcription of a number of genes in a variety of cancers [8,37]. These genes are relevant to cancer cell division, to apoptosis, to invasiveness, and to tumor-relevant angiogenesis in a large panel of carcinomas of various organs. We have reviewed here the evidence for the trophic action of T4 on various forms of thyroid cancer. It is clear, however, that additional investigation is needed to confirm the extent of such action of T4. It is also of some importance to deal with the possibility that thyroid hormone analogues may act via integrin αvβ3 to alter thyroid tumor radiosensitivity as they have been shown to do in other forms of cancers [41,42]. Also needed are studies that assess the possibility of effects of T4 on anaplastic thyroid carcinomas.

When differentiated thyroid carcinomas remain clinically active, despite full suppression of host TSH, we would suggest the possibility that the tumor is now T4-responsive [3]. A therapeutic option in this setting is induction of euthyroid hypothyroxinemia that we have tested clinically in a variety of advanced, T4-responsive (non-thyroid) cancers [43]. The limited genetic data we have reviewed in the current paper indicates a need for comparing genotypic information from differentiated, but now aggressive, thyroid cancers that (1) are and are not TSH-responsive and (2) are and are not T4-responsive in vitro. Genotyping of aspiration biopsies might then be useful in considering interruption of T4-suppression of TSH. 

We have noted above that rT3—another thyroid hormone analogue thought to have little or no biological activity—is capable of stimulating cancer cell proliferation [11]. This proliferative effect has not yet been sought experimentally in differentiated thyroid carcinoma. However, the Type 3 deiodinase that generates rT3 from T4 [44] is present in papillary thyroid carcinoma cells [45]. 

Finally, we have also pointed out in the current review that leptin may be a trophic factor for papillary thyroid carcinoma, a form of cancer known to express the leptin receptor [5]. These interesting preclinical studies are of potential relevance to suboptimal clinical response of well-differentiated papillary thyroid cancer to TSH suppression in overweight patients. The genes whose expression was affected by leptin include those linked to angiogenesis and invasiveness.
